# Supplementary material for: TRAP1 S-nitrosylation as a model of population-shift mechanism to study the effects of nitric oxide on redox-sensitive oncoproteins
Source: Cell Death Dis. 2023 Apr 21;14(4):284. doi: 10.1038/s41419-023-05780-6 (PMC10121659; doi:10.1038/s41419-023-05780-6)
Supplement: Supplementary file 1 — Supplementary Figure S1 [file 41419_2023_5780_MOESM1_ESM.pdf]

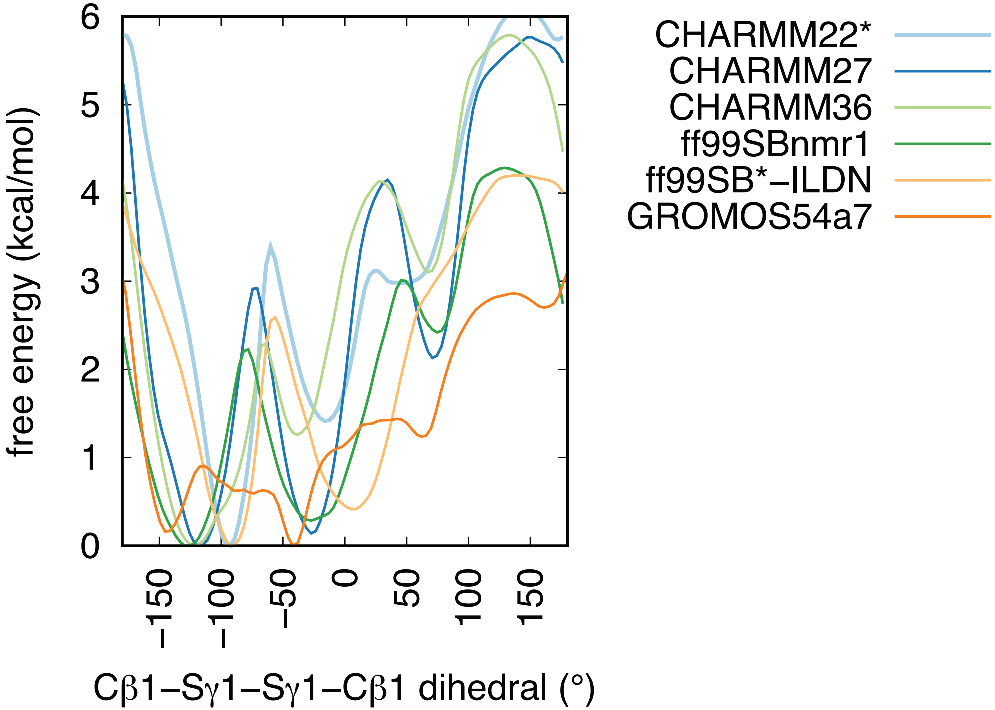

Figure S1 Mono-dimensional free energy profiles for the Cβ-Sγ-Sγ-Cβ dihedral calculated on the metadynamics of *Danio rerio* TRAP1$_{311\text{-}567}$ with both SNO site and the proximal cysteine in their reduced form. We used eight different MD force fields. We observed that the different force fields overall captured the same main minima.
