# Supplementary material for: TRAP1 S-nitrosylation as a model of population-shift mechanism to study the effects of nitric oxide on redox-sensitive oncoproteins
Source: Cell Death Dis. 2023 Apr 21;14(4):284. doi: 10.1038/s41419-023-05780-6 (PMC10121659; doi:10.1038/s41419-023-05780-6)
Supplement: Supplementary file 2 — Supplementary Figure S2 [file 41419_2023_5780_MOESM2_ESM.pdf]

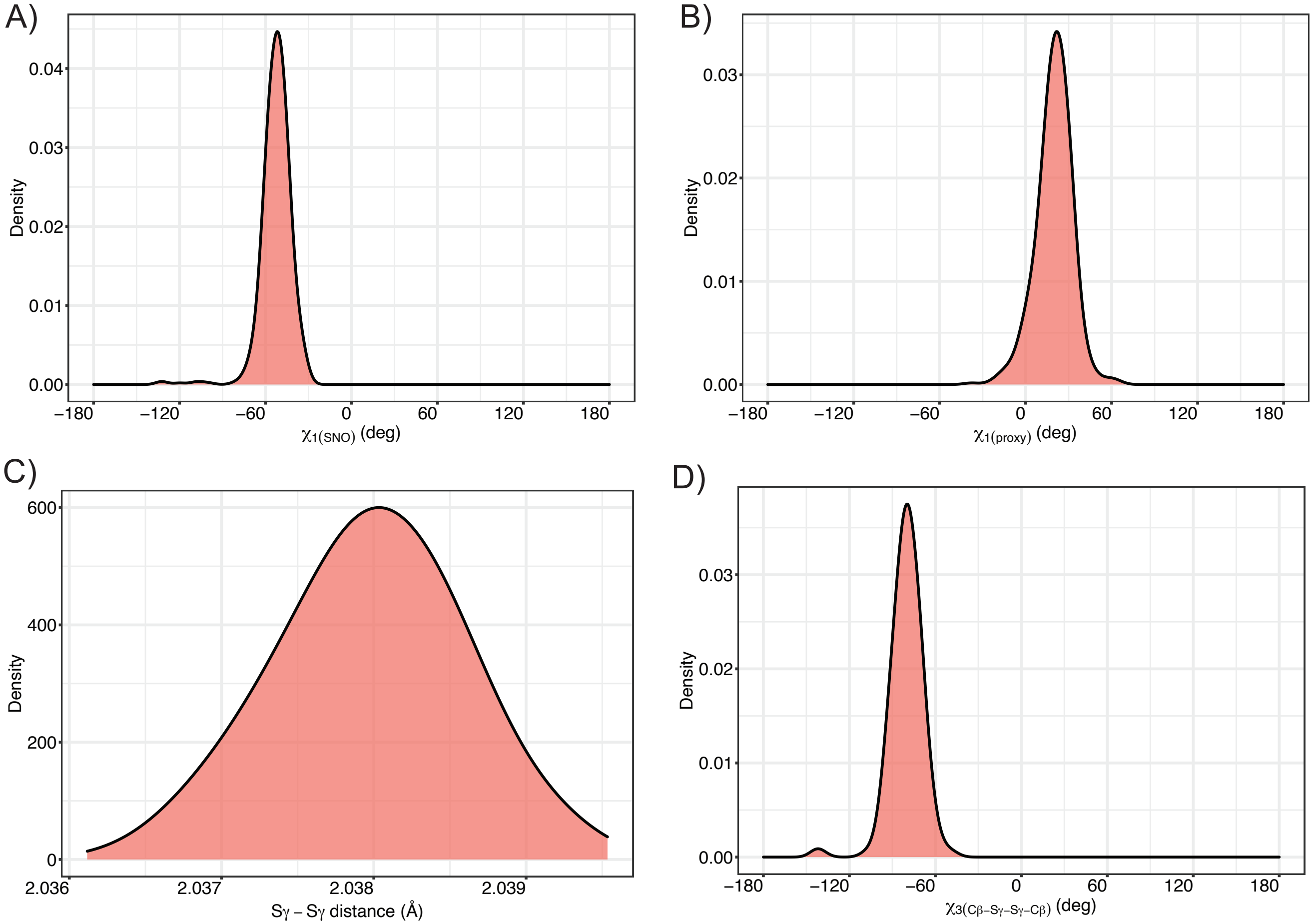

Figure S2 Analysis of the unbiased MD simulation of TRAP1$_{227\text{-}438}$ with a disulfide bridge between C527 and C542. We analyzed the collective variables used in metadynamics on a reference one-μs unbiased MD simulation of the oxidized form of TRAP1$_{311\text{-}567}$ with a disulfide bridge formed between C527, the S-nitrosylation site, and proximal C542. The plots show the distribution of the values calculated for the collective variables A) $\chi 1$ dihedral of the S-nitrosylation site C527 ($\chi_{1(SNO)}$), B) $\chi 1$ dihedral of the proximal C542 ($\chi_{1(proxy)}$), C) the distance between their sulfur atoms (Sγ-Sγ distance), D) the dihedral angle Cβ-Sγ-Sγ-Cβ. We observed that the Sγ-Sγ distance is approximately 2.03 Å. The Cβ-Sγ-Sγ-Cβ dihedral has a peak at -90 degrees which is common for disulfide bridges. In addition, we observed minus and plus states for the $\chi 1$ of the S-nitrosylation and proximal cysteine, respectively.
