# Supplementary material for: TRAP1 S-nitrosylation as a model of population-shift mechanism to study the effects of nitric oxide on redox-sensitive oncoproteins
Source: Cell Death Dis. 2023 Apr 21;14(4):284. doi: 10.1038/s41419-023-05780-6 (PMC10121659; doi:10.1038/s41419-023-05780-6)
Supplement: Supplementary file 3 — Supplementary Figure S3 [file 41419_2023_5780_MOESM3_ESM.pdf]

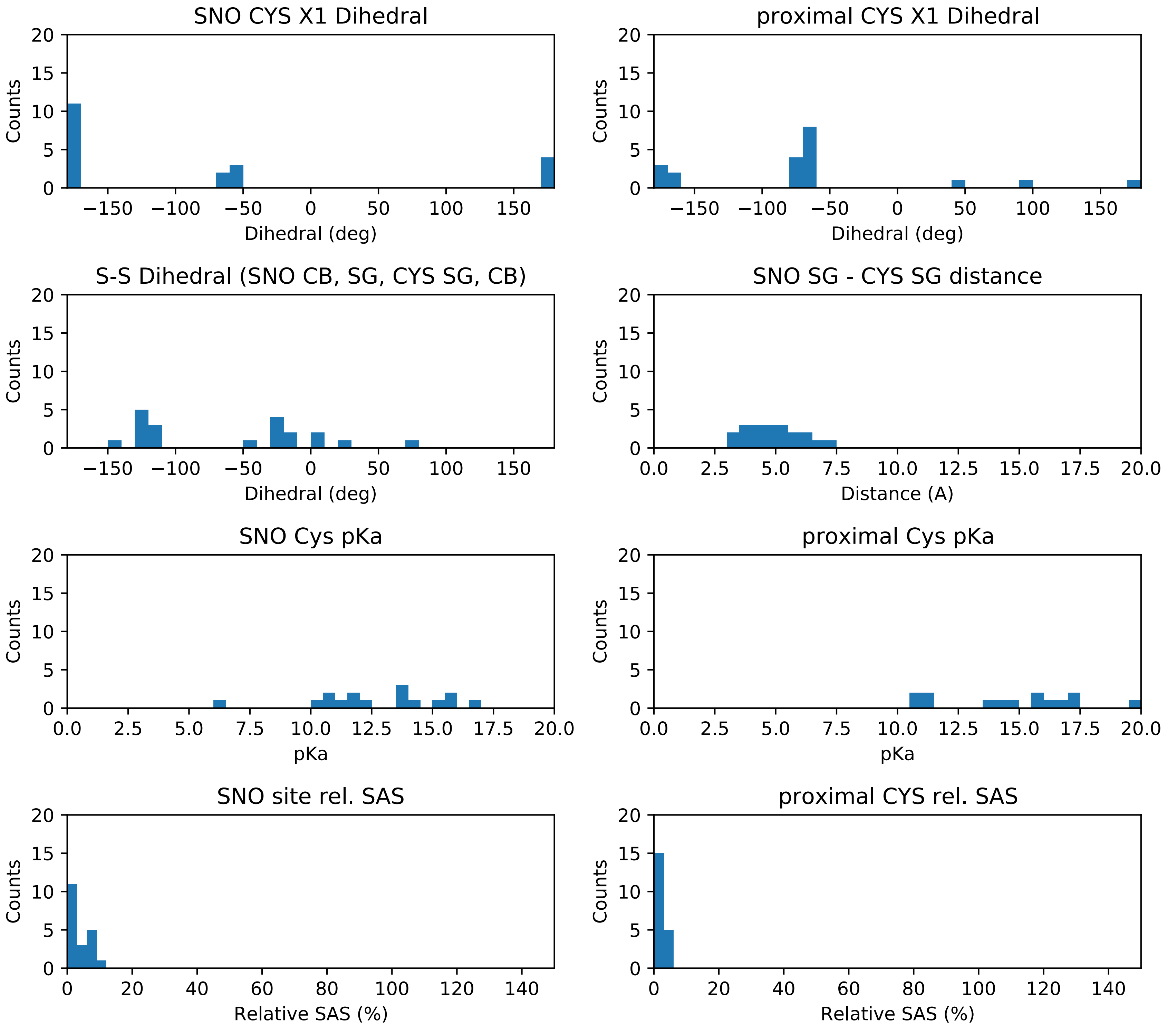

Figure S3: Distributions of values for properties of interest in the CABSflex ensemble of TRAP1, as calculated by SNOmodels.
