# Supplementary figures and images for: TRAP1 S-nitrosylation as a model of population-shift mechanism to study the effects of nitric oxide on redox-sensitive oncoproteins
Source: Cell Death Dis. 2023 Apr 21;14(4):284. doi: 10.1038/s41419-023-05780-6 (PMC10121659; doi:10.1038/s41419-023-05780-6)

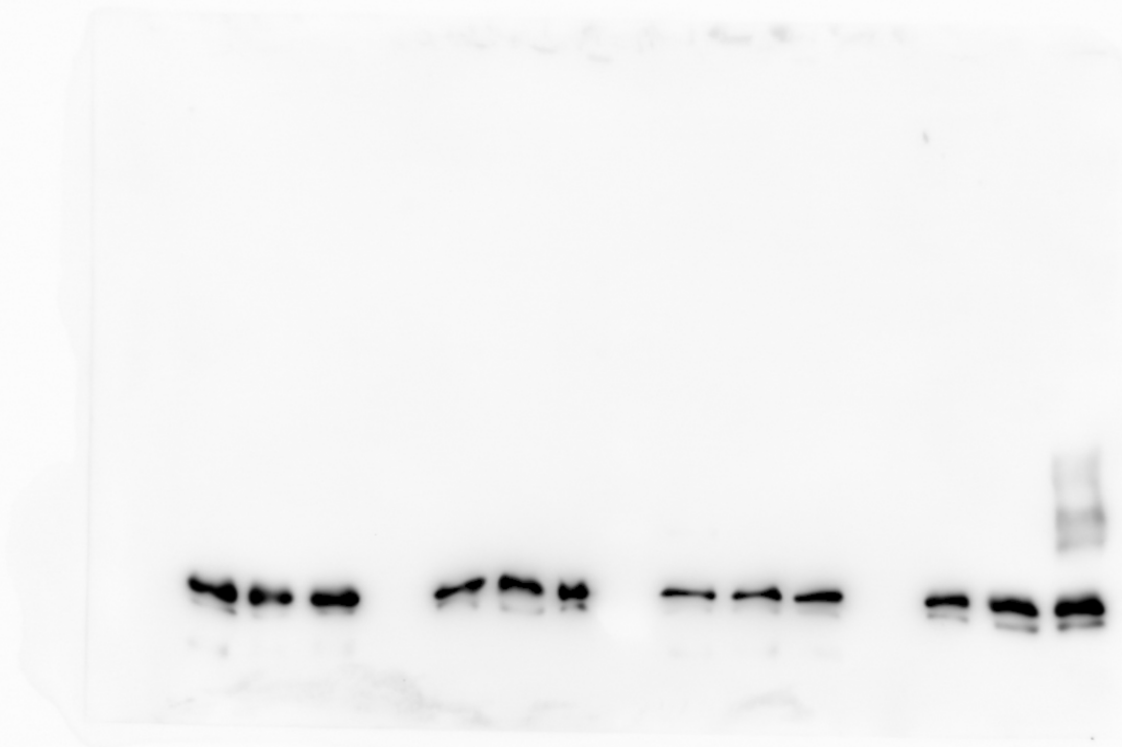

**Figure S4:** Original data file for western blots

Supplement: Supplementary file 4 — Original Data File [file 41419_2023_5780_MOESM4_ESM.pdf]
